# A descriptive, retrospective single‐centre study of air‐leak syndrome in intensive care unit patients with COVID‐19

**DOI:** 10.1111/aas.14582

**Published:** 2025-02-12

**Authors:** Alice Löwing Jensen, Jacob Litorell, Jonathan Grip, Martin Dahlberg, Eva Joelsson‐Alm, Sandra Jonmarker

**Affiliations:** ^1^ Department of Anaesthesia and Intensive Care Södersjukhuset Stockholm Sweden; ^2^ Department of Clinical Science, Intervention and Technology, CLINTEC Karolinska Institutet Stockholm Sweden; ^3^ Function Perioperative Medicine and Intensive Care Karolinska University Hospital Stockholm Sweden; ^4^ Department of Surgery Södersjukhuset Stockholm Sweden; ^5^ Department of Clinical Science and Education, Södersjukhuset Karolinska Institutet Stockholm Sweden

**Keywords:** air‐leak syndrome, barotrauma, COVID‐19, intensive care

## Abstract

**Background:**

Acute respiratory failure is the predominant presentation of intensive care unit (ICU) patients with COVID‐19, and lung protective strategies are recommended to mitigate additional respiratory complications such as air‐leak syndrome. The aim of this study is to investigate the prevalence, type, and timing of air‐leak syndrome with regards to associated factors and patient outcome in patients with COVID‐19 in ICUs at a large Swedish emergency hospital.

**Methods:**

This retrospective study included all adult patients admitted to an ICU for COVID‐19‐related respiratory failure at Södersjukhuset between March 6, 2020, and June 6, 2021. Primary outcomes were proportion of patients diagnosed with air‐leak syndrome and its different types of manifestations, and timing of diagnoses in relation to ICU admission and initiation of invasive ventilation. Secondary outcomes included the highest level of respiratory support prior to the diagnosis of air‐leak syndrome, patient characteristics and treatment variables associated with air‐leak syndrome, and 90‐day mortality for patients with air‐leak syndrome compared to those without.

**Results:**

Out of a total of 669 patients, 81 (12%) were diagnosed with air‐leak syndrome. Air‐leak syndrome manifested as pneumomediastinum (PMD) (*n* = 58, 72%), pneumothorax (PTX) (*n* = 43, 53%), subcutaneous emphysema (SCE) (*n* = 28, 35%) and pneumatocele (PC) (*n* = 4, 4.9%). Air‐leak syndrome was diagnosed at a median of 14 days (IQR 6–22) after ICU admission and 12 days (IQR 6–19) following the initiation of invasive ventilation. The highest respiratory support prior to diagnosis was invasive ventilation (IV) in 64 patients (79%), non‐invasive ventilation in two patients (2.5%), and low‐ or high‐flow oxygen in 15 patients (19%). Multiple logistic regression showed that pulmonary disease at baseline (OR 1.87, 95% CI 1.07–3.25), a lower body mass index (OR 0.95, 95% CI 0.9–0.99), admission later compared with earlier in the pandemic (OR 3.89, 95% CI 2.14–7.08), and IV (OR 3.92, 95% CI 2.07–7.44) were associated with an increased risk of air‐leak syndrome. Compared with patients not diagnosed with air‐leak syndrome, patients with air‐leaks had a higher mortality at 90 days after ICU admission, 46% versus 26% (*p* <.001). However, the mortality rate differed with different air‐leak manifestations, 47% for PMD, 47% for PTX, 50% for the combination of both PMD and PTX and 0% in patients with only SCE and/or PC, respectively.

**Conclusion:**

In 669 ICU patients with COVID‐19, 12% had one or more manifestations of air‐leak syndrome. Notably, PMD, rather than PTX, was the most common manifestation, suggesting a potentially distinctive feature of COVID‐19‐related air‐leak syndrome. Further research is needed to determine whether COVID‐19 involves different pathophysiological or iatrogenic mechanisms compared with other critical respiratory conditions.

**Registration of Clinical Trial:**

Clinicaltrials.gov, identifying number, NCT05877443.

**Editorial Comment:**

This single‐centre cohort study of air leakage into soft tissue in ventilated COVID cases presents findings for associated factors and clinical manifestations, including with different COVID‐19 periods and treatments.

## 1 INTRODUCTION

Since 2020, COVID‐19 has spread across the globe, affecting individuals with a varying disease burden, from asymptomatic carriers to critical illness causing death.^1^, ^2^ Most patients admitted to an intensive care unit (ICU) have bilateral interstitial pneumonitis causing acute respiratory failure. Treatment of ICU patients requiring mechanical ventilation includes lung‐protective ventilation strategies, aiming to prevent ventilator‐induced lung injury (VILI).^3^, ^4^, ^5^, ^6^ One type of VILI is air‐leak syndrome, referring to a rupture of alveoli followed by release or dissection of air into extra alveolar spaces.^7^ Before the COVID‐19 pandemic, the incidence of air‐leak syndrome in patients with respiratory failure meeting the definition of acute respiratory distress syndrome (ARDS) was reported to be 6%–11%.^3^, ^7^ For patients with COVID‐19, studies have reported a higher incidence of air‐leak syndrome with atypical manifestations.^8^, ^9^, ^10^, ^11^, ^12^ This aligns closely with our clinical experience, particularly in patients with the most severe illness. Therefore, our aim was to investigate the prevalence, type, and timing of air‐leak syndrome in ICU patients with respiratory failure due to COVID‐19 in a large Swedish emergency hospital. Additionally, we aimed to investigate the level of respiratory support patients received before diagnosis, how various patient characteristics and treatment variables were associated with the risk of developing air‐leak syndrome, and whether the diagnosis affected mortality risk.

## 2 METHODS

### 2.1 Study overview and participants

This retrospective, descriptive study included all adult patients admitted to an ICU with respiratory failure due to COVID‐19 at Södersjukhuset, Stockholm, Sweden, between March 6, 2020, and June 6, 2021. The diagnosis of Severe Acute Respiratory Syndrome Corona Virus 2 (SARS‐CoV‐2) was confirmed by reverse transcription polymerase chain reaction test. Patients with air‐leak syndrome secondary to an invasive procedure (e.g., PTX during central line insertion or minor air accumulation in the operating area following tracheostomy) were not included in the air‐leak syndrome group. If a patient was admitted more than once, the subsequent admissions were excluded.

This study was retrospectively registered at ClinicalTrials.gov (identification number NCT05877443) and received approval from the Swedish Ethical Review Authority (Dnr: 2020–01302, approved on 2020‐04‐03; amendment 2020–02890, approved on 2020‐06‐18), with a waiver for the requirement of informed consent. The reporting of the study adheres to the Strengthening The Reporting of Observational Studies in Epidemiology (STROBE) guidelines.^13^

Data regarding baseline characteristics (age, sex, BMI, cardiovascular, and pulmonary comorbidities), air‐leak syndrome including type, initial PaO_2_/FiO_2_‐index on admission to the ICU (value accepted if within 48 h of admission), admission period (early: March 2020–August 2020, later: September 2020–June 2021), level of respiratory support, tracheostomy, use of prone positioning, glucocorticoid therapy, and mortality at 90 days after admission to the ICU were extracted from electronical health records. The level of respiratory support was categorized into three groups based on the highest level of support received during the ICU stay: low‐flow oxygenation (LFO)/high‐flow nasal oxygenation (HFNO), non‐invasive ventilation (NIV), and invasive ventilation (IV). Glucocorticoid therapy was categorized into three groups: patients who did not receive glucocorticoids during their ICU stay, patients who received glucocorticoids but did not meet the criteria equivalent to the dose prescribed in the RECOVERY trial (defined as ≥6 mg of betamethasone or an equivalent dose per day, initiated within 7 days of hospital admission, and continued for at least 1 day), and patients who received doses meeting the criteria equivalent to the dose prescribed in the RECOVERY trial.^14^

### 2.2 Outcomes

Primary outcomes were the proportion of patients diagnosed with air‐leak syndrome and its different types of manifestations, and the timing of diagnoses in relation to ICU admission and initiation of invasive ventilation. Air‐leak syndrome was defined as the presence of pneumomediastinum (PMD), pneumothorax (PTX), subcutaneous emphysema (SCE), and/or pneumatocele (PC) verified by chest x‐ray (CXR) or computed tomography (CT). A diagnosis by ultrasound without confirmation by CXR or CT was not considered sufficient. All radiological images were reviewed by two separate radiologists, as is standard procedure at Södersjukhuset. The time of diagnosis of air‐leak syndrome was described as both in relation to ICU admission and from initiation of IV for patients needing this level of respiratory support.

Secondary outcomes were associations between the highest prior respiratory support, patient characteristics and treatment variables and the diagnosis of air‐leak syndrome, as well as the comparison of 90‐day mortality between patients with and without air‐leak syndrome. Patients' characteristics investigated included age, sex, BMI, cardiovascular comorbidities (defined as hypertension, ischemic heart disease, heart failure, atrial fibrillation, atrial flutter, peripheral vascular disease, cerebrovascular disease), pulmonary comorbidities (defined as asthma, chronic obstructive lung disease, restrictive lung disease) and PaO_2_/FiO_2_ at ICU admission. Treatment variables investigated included time period of admission and highest ventilatory support prior to diagnosis.

### 2.3 Statistical analysis

Continuous variables were described as median with interquartile range and compared by applying the Wilcoxon rank‐sum test. Categorical variables were described as proportions and compared using Pearson's chi‐square test or Fisher's exact test, as appropriate.

A multivariable logistic regression model using direct selection was performed to identify variables associated with the diagnosis of air‐leak syndrome. The variables of age (continuous), sex (male/female), BMI (continuous), cardiovascular comorbidity (yes/no), pulmonary comorbidity (yes/no), PaO_2_/FiO_2_ (continuous), time period (early/later), highest level of respiratory support (LFO and HFNO/NIV/IV), and diagnosis of air‐leak syndrome (yes/no) were included. The use of glucocorticoids was not included in the model as we suspected collinearity with time period. To ensure an adequate sample size and avoid overfitting, the model was designed so that the number of events per variable was at least 10. The model was tested for multicollinearity using Variance Inflation Factor accepting values ≤5. Splines were used to assess monotonicity and linearity. Results were presented as odds ratio (OR) and 95% confidence interval (CI). Kaplan–Meier curves were used to estimate the cumulative risk of death over 90 days. No imputation model was used to replace data and, therefore, patients with missing data were excluded.

Statistical significance was defined as a two‐sided *p*‐value <0.05. All statistical analyses were performed using R version 4.3.0. (R Core Team (2023), Foundation for Statistical Computing, Vienna, Austria).

## 3 RESULTS

**FIGURE 1:**
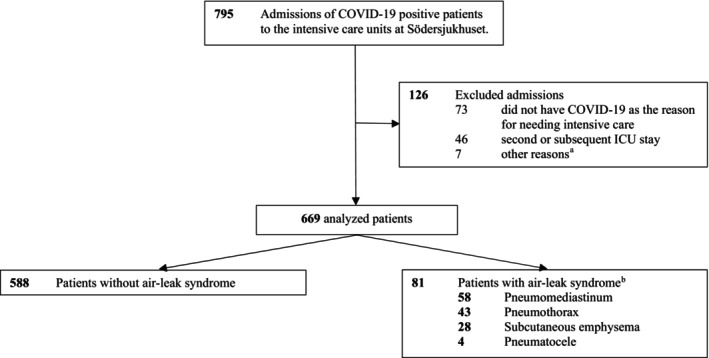
Flow chart of included patients with respiratory failure due to COVID‐19 admitted to the intensive care units at Södersjukhuset, Stockholm, Sweden, March 2020 to June 2021. ^a^Five patients were excluded as PCR was negative for COVID‐19, and two patients were excluded due to PCR not being available as they were transferred from another hospital. ^b^There were 34 patients who had more than one manifestation of air‐leak syndrome.

A total of 669 patients admitted to an ICU, between March 6, 2020, and June 6, 2021, at Södersjukhuset, Stockholm, Sweden, were included (Figure 1). Table 1 reports baseline, treatment and outcome characteristics in patients with versus those without diagnosis of air‐leak syndrome. Of the patients included, 81 (12%) were diagnosed with air‐leak syndrome. The diagnosis was confirmed with CT in 45 patients (56%) and with CXR in 36 patients (44%). There was no statistically significant difference in age or sex between patients with and without diagnosis of air‐leak syndrome. BMI and PaO_2_/FiO_2_ on admission were lower in the air‐leak syndrome group. Cardiovascular comorbidity did not differ between groups, but pulmonary diseases were more common in the group with air‐leak syndrome. Patients with air‐leak syndrome were more likely to be treated with IV and receive a tracheostomy at some point during their ICU stay. A higher proportion of patients admitted during the later stages of the pandemic were diagnosed with air‐leak syndrome compared with patients admitted during the early time period. A total of 311 of 669 patients were proned at least once, and this was a more common treatment among patients with air‐leak syndrome as compared to patients without air‐leak (68% and 44%, *p* <.001). Patients with air‐leak syndrome received glucocorticoids to a greater extent during their ICU stay compared to those without air‐leak syndrome. Mortality at 90 days after ICU admission was higher in patients with air‐leak syndrome compared to patients without air‐leak syndrome (46% vs. 26%, *p* <.001), seen in Table 1.

**TABLE 1:** Patient baseline, treatment, and outcome characteristics.

	Overall	No air‐leak syndrome	Air‐leak syndrome	*p*‐value^a^
	*N* = 669	*N* = 588	*N* = 81	
Age, median (IQR), years	64 (55, 72)	64 (54, 73)	64 (59, 70)	0.5
Sex‐female	191 (29)	172 (29)	19 (23)	0.3
BMI, median (IQR), kg/m	28 (25, 32) (*n* = 661)	28 (25, 33) (*n* = 580)	27 (24, 31)	0.018
Cardiovascular disease^b^	374 (56)	334 (57)	40 (49)	0.2
Pulmonary disease^c^	164 (25)	136 (23)	28 (35)	0.025
PaO_2_/FiO_2_, median (IQR), kPa/fraction	11 (9,14) (*n* = 666)	11 (9, 15) (*n* = 585)	10 (8, 13)	0.038
Use of prone position	311 (46)	256 (44)	55 (68)	<.001
Glucocorticoids				<.001
No treatment	139 (21)	135 (23)	4 (4.9)	
Treatment but did not meet the criteria equivalent to the dose prescribed in the RECOVERY trial^d^	68 (10)	63 (11)	5 (6.2)	
Treatment according to the criteria equivalent to the dose prescribed in the RECOVERY trial^d^	462 (69)	390 (66)	72 (89)	
Most invasive form of ventilation				<.001
LFO/HFNO	219 (33)	208 (36)	11 (13)	
NIV	103 (15)	101 (17)	2 (3)	
IV	347 (52)	279 (47)	68 (84)	
Time period				<.001
Early^e^	264 (39)	247 (42)	17 (21)	
Later^f^	405 (61)	341 (58)	64 (79)	
Tracheostomy during ICU stay	166 (25)	118 (20)	48 (59)	<.001
Mortality at 90 days post ICU admission	191 (29)	154 (26)	37 (46)	<.001

*Note*: Patient baseline, treatment, and outcome characteristics. Values are expressed as no. (%) unless otherwise indicated. Data are complete unless indicated by number of patients.

Abbreviations: BMI, body mass index; HFNO, high flow nasal oxygen; ICU, intensive care unit; IV, invasive ventilation; LFO, low flow oxygen; NIV, non‐invasive ventilation; PaO_2_/FiO_2_ Partial pressure of oxygen/Fraction of inspired oxygen.

^a^ Wilcoxon rank sum test; Pearson's Chi‐squared test; Fisher's exact test.

^b^ Cardiovascular disease is defined as a diagnosis of hypertension, ischemic heart disease, heart failure, atrial fibrillation, atrial flutter, peripheral vascular disease, and cerebrovascular disease.

^c^ Pulmonary disease is defined as asthma, chronic obstructive lung disease, and restrictive lung diseases.

^d^ Criteria defined as ≥6 mg of betamethasone or an equivalent dose per day, initiated within 7 days of hospital admission, and continued for at least 1 day.

^e^ March 2020 to August 2020.

^f^ September 2020 to June 2021.

**FIGURE 2:**
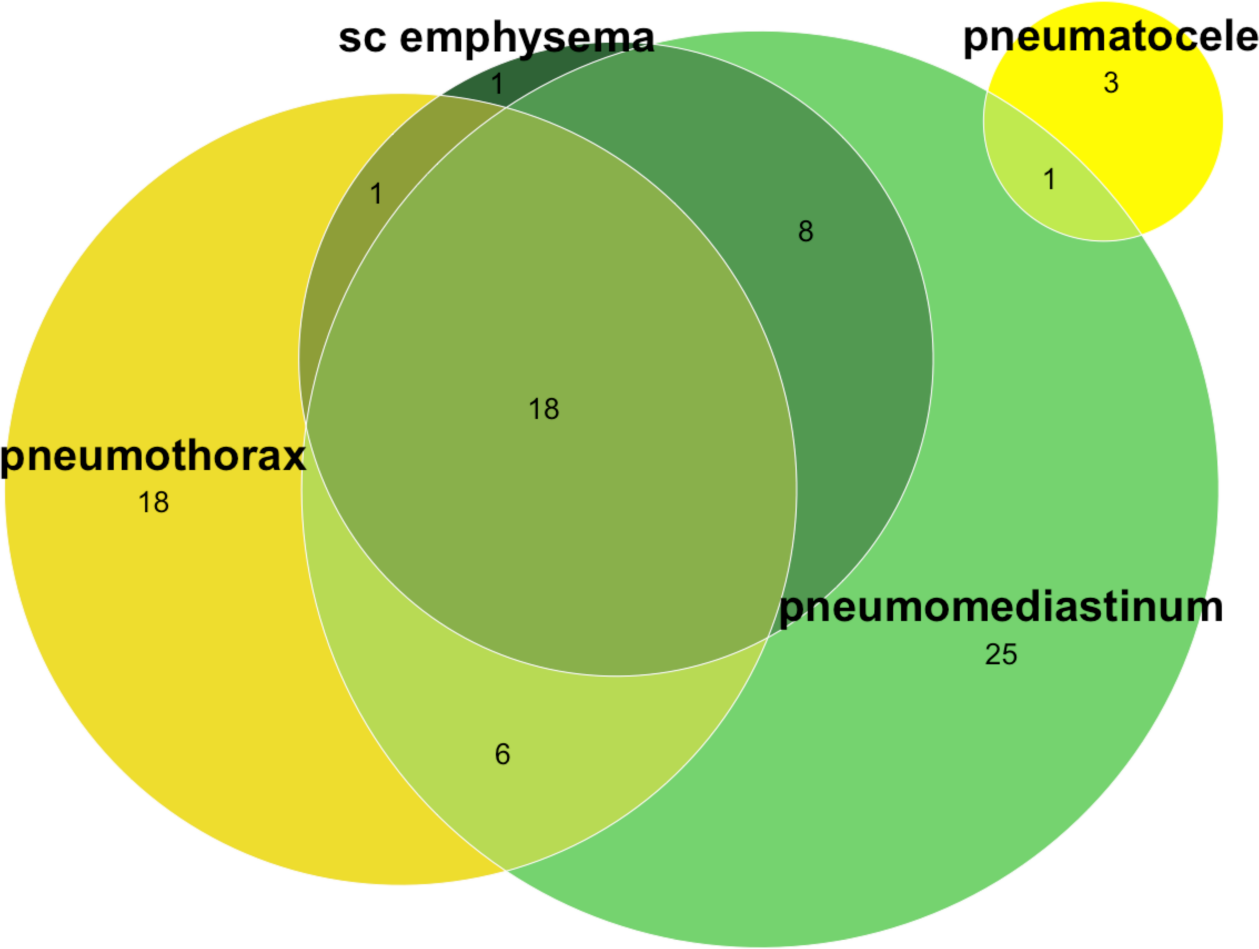
Illustration of the types and combinations of air‐leak syndrome among 81 patients with respiratory failure due to COVID‐19, Södersjukhuset, Stockholm, Sweden, March 2020 to June 2021. Air‐leak syndrome was defined as pneumomediastinum, pneumothorax, subcutaneous emphysema or pneumatocele, verified by radiology.

**FIGURE 3:**
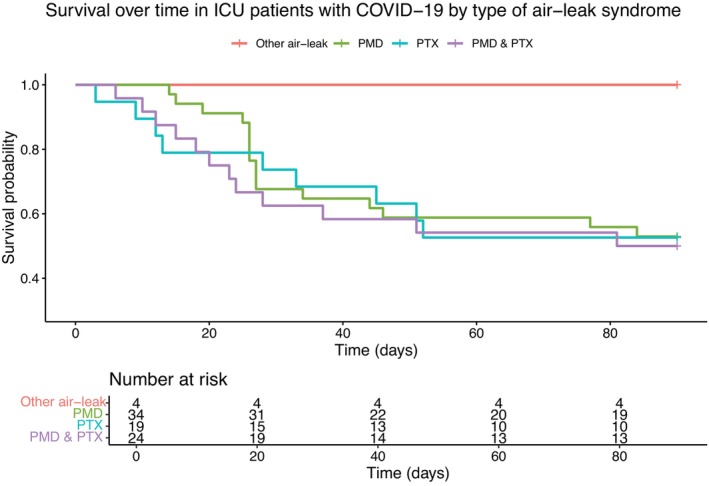
Kaplan–Meier plots of survival over time from intensive care unit (ICU) admission among 81 ICU patients with respiratory failure due to COVID‐19 and air‐leak syndrome, categorized by type of air‐leak syndrome, Södersjukhuset, Stockholm, Sweden, March 2020 to June 2021. Air‐leak syndrome was defined as pneumomediastinum, pneumothorax, subcutaneous emphysema or pneumatocele, verified by radiology. Patients were followed for 90 days or until death, whichever occurred first. PMD, pneumomediastinum; PTX, pneumothorax.

**FIGURE 4:**
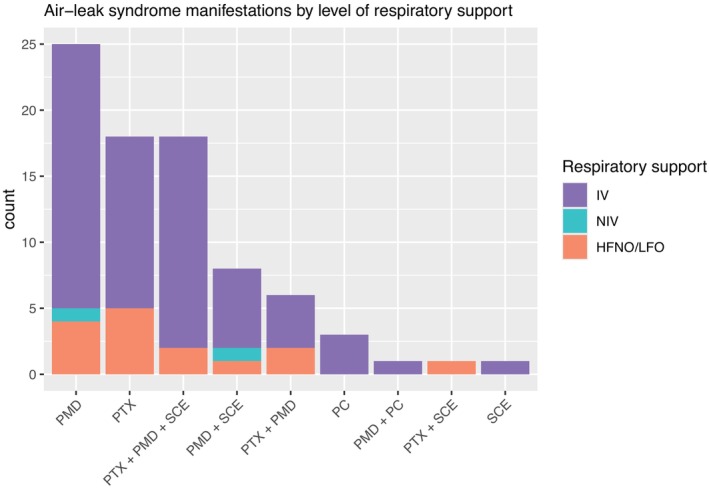
Bar chart illustrating the manifestations of air‐leak syndrome among 81 intensive care unit (ICU) patients with respiratory failure due to COVID‐19 by the highest level of respiratory support prior to air‐leak syndrome, admitted to the ICUs at Södersjukhuset, Stockholm, Sweden, March 2020 to June 2021. HFNO/LFO, high‐flow nasal oxygen/low‐flow oxygen; IV, invasive ventilation; NIV, non‐invasive ventilation; PC, pneumatocele; PMD, pneumomediastinum; PTX, pneumothorax; SCE, subcutaneous emphysema.

Air‐leak syndrome manifested as PMD (*n* = 58, 72%), PTX (*n* = 43, 53%), SCE (*n* = 28, 35%), and PC (*n* = 4, 4.9%), see Table 2 and Figure 2. A majority of patients (*n* = 47, 58%) had an isolated manifestation of air‐leak syndrome, 16 patients (20%) had two manifestations and 18 patients (22%) had three manifestations. Mortality at 90‐days by type of air‐leak syndrome was 47% (16/34) in patients with PMD (with or without SCE/PC), 47% (9/19) in patients with PTX (with or without SCE/PC), 50% (12/24) in patients with both PMD and PTX (with or without SCE/PC), and 0% (0/4) in patients with only SCE and/or PC (Figure 3). Six patients (7.4%) were diagnosed with manifestations of air‐leak syndrome before ICU admission, 69 (85%) within 28 days after ICU admission and six (7.4%) more than 28 days after ICU admission. Median time from ICU admission to air‐leak syndrome was 14 days (IQR 6–22) and from intubation to air‐leak syndrome was 12 days (IQR 3–19), not including patients with air‐leak syndrome prior to ICU admission/intubation (Table 2). Prior to diagnosis of air‐leak syndrome, 64 patients (79%) had been treated with IV, two (2.5%) had received NIV as the highest level of respiratory support and 15 (19%) had only received HFNO or LFO, as shown in Figure 4. Thirty‐two (40%) of all patients with air‐leak had a tracheostomy before diagnosis (Table 2). Of the 81 patients with air‐leak syndrome, 27 had a surgical procedure to drain the air and all these patients had the air‐leak type of PTX.

**TABLE 2:** Type, number, timing and most invasive ventilation (IV) for patients with air‐leak syndrome.

	Air‐leak syndrome
	*N* = 81
Type of air‐leak syndrome	
Pneumomediastinum	58 (72)
Pneumothorax	43 (53)
Subcutaneous emphysema	28 (35)
Pneumatocele	4 (4.9)
Number of manifestations	
1	47 (58)
2	16 (20)
3	18 (22)
Air‐leak syndrome, timing	
Before ICU admission	6 (7.4)
Within 28 days of ICU admission	69 (85)
After 28 days of ICU admission	6 (7.4)
Time to air‐leak syndrome from ICU admission, median (IQR), days^a^	14 (6, 22)
Time to air‐leak syndrome from initiation of invasive ventilation, median (IQR), days^b^	12 (6, 19)
Most invasive form of ventilation prior to air‐leak syndrome	
LFO/HFNO	15 (19)
Non‐invasive	2 (2.5)
Invasive	64 (79)
Tracheostomy prior to air‐leak syndrome	32 (40)

*Note*: Type, number, timing, and most invasive ventilation for patients with air‐leak syndrome. Values are expressed as no. (%) unless otherwise indicated.

Abbreviations: HFNO, high flow nasal oxygen; ICU, intensive care unit; IV, invasive ventilation; LFO, low flow oxygen; NIV, non‐invasive ventilation.

^a^ For patients admitted to ICU prior to, or at the same date, as the diagnosis of air‐leak syndrome.

^b^ For patients with invasive ventilation started prior to, or at the same date, as the diagnosis of air‐leak syndrome.

Table 3 details the distribution of respiratory support, glucocorticoid therapy and air‐leak syndrome by time period. During the early time period, 61% of patients received IV, compared to 46% in the later time period. Nearly all patients in the later time period (403/405) received treatment with glucocorticoids equivalent to the RECOVERY‐trial, compared to only 22% in the early time period. Air‐leak syndrome was more commonly diagnosed in the later, compared to the early, time period (16% vs. 6.4%).

**TABLE 3:** Patient characteristics, respiratory support, glucocorticoids, and 90‐day mortality by time period.

		Early time period^a^			Later time period^b^		
	All patients (*n* = 669)	All patients early time period (*n* = 264)	No air‐leak syndrome (*n* = 247)	Air‐leak syndrome (*n* = 17)	All patients later time period (*n* = 405)	No air‐leak syndrome (*n* = 341)	Air‐leak syndrome (*n* = 64)
Age, median (IQR), years	64 (55, 72)	61 (52, 71)	61 (52, 71)	66 (63, 68)	65 (58, 74)	66 (57, 74)	64 (58, 70)
Sex‐female	191 (29)	60 (23)	57 (23)	3 (18)	131 (32)	115 (34)	16 (25)
BMI, median (IQR), kg/m	28 (25, 32) (*n* = 661)	28 (25, 32) (*n* = 259)	28 (25, 32) (*n* = 242)	25 (24, 27)	29 (25, 33) (*n* = 402)	29 (25, 33) (*n* = 338)	28 (24, 31)
PaO_2_/FiO_2_, median (IQR), kPa/fraction	11 (9, 14) (*n* = 666)	12 (10, 15) (*n* = 264)	12 (10, 15) (*n* = 245)	11 (8, 14)	11 (9, 14) (*n* = 404)	11 (9, 14) (*n* = 340)	10 (8, 12)
Most invasive form of respiratory support							
LFO/HFNO	219 (33)	86 (33)	85 (34)	1 (6)	133 (33)	123 (36)	10 (16)
NIV	103 (15)	17 (6)	17 (7)	0 (0)	86 (21)	84 (24)	2 (3)
IV	347 (52)	161 (61)	145 (59)	16 (94)	186 (46)	134 (39)	52 (81)
Glucocorticoids							
No glucocorticoids	139 (21)	137 (52)	133 (54)	4 (24)	2 (0.5)	2 (1)	0 (0)
Glucocorticoids not meeting criteria for COVID‐19 treatment^c^	68 (10)	68 (26)	63 (26)	5 (29)	0 (0)	0 (0)	0 (0)
Glucocorticoids meeting criteria for COVID‐19 treatment^c^	462 (69)	59 (22)	51 (21)	8 (47)	403 (99)	339 (99)	64 (100)
90‐day mortality	191 (29)	68 (26)	61 (25)	7 (41)	123 (30)	93 (27)	30 (47)

*Note*: Patient characteristics, respiratory support, glucocorticoids, and 90‐day mortality by time period. Values are expressed as no. (%) unless otherwise indicated. Data are complete unless indicated by number of patients.

Abbreviations: BMI, body mass index; HFNO, high flow nasal oxygen; IV, invasive ventilation; LFO, low flow oxygen; NIV, non‐invasive ventilation; PaO_2_/FiO_2_, Partial pressure of oxygen/Fraction of inspired oxygen.

^a^ March 2020 to August 2020.

^b^ September 2020 to June 2021.

^c^ Criteria defined as ≥6 mg of betamethasone or an equivalent dose per day, initiated within 7 days of hospital admission, and continued for at least 1 day.

Multivariable logistic regression analysis showed that air‐leak syndrome was associated with pulmonary disease at baseline (OR 1.87, 95% CI 1.07–3.25), a lower BMI (OR 0.95, 95% CI 0.9–0.99), admission during the later time period (OR 3.89, 95% CI 2.14–7.08) and IV (OR 3.92, 95% CI 2.07–7.44), seen in Table 4. The associations remained stable when excluding nine patients for whom the absence of air‐leak had not been confirmed on CXR/CT after intubation but prior to the CXR/CT confirming the diagnosis.

**TABLE 4:** Results of multivariable regression.

	Analysis with all patients (*n* = 669)			Analysis when excluding nine patients without CXT or CT confirming the absence of air‐leak after intubation (*n* = 660)		
	OR	95% CI	*p*‐value	OR	95% CI	*p*‐value
Age	1.01	0.99–1.04	0.30	1.01	0.99–1.03	0.25
Male sex	1.57	0.86–2.85	0.14	1.5	0.82–2.86	0.18
BMI	0.95	0.90–1.00	0.04	0.95	0.90–1.00	0.04
Cardiovascular disease^a^	0.66	0.38–1.14	0.14	0.63	0.36–1.12	0.12
Pulmonary disease^b^	1.87	1.07–3.25	0.03	1.84	1.03–3.28	0.04
PaO_2_/FiO_2_	1.00	0.96–1.05	0.96	1.00	0.96–1.05	0.77
Later time period^c^	3.89	2.14–7.08	<.001	3.84	2.04–7.22	<.001
NIV prior to air‐leak syndrome^d^	0.23	0.05–1.05	0.06	0.23	0.05–1.07	0.06
IV prior to air‐leak syndrome^d^	3.92	2.07–7.44	<.001	3.43	1.79–6.57	<.001

*Note*: Results of the multivariable analysis showing odds ratio between air‐leak syndrome and variables of interest.

Abbreviations: BMI, body mass index; CI, confidence interval; IV, invasive ventilation; NIV, non‐invasive ventilation; OR, odds ratio; PaO_2_/FiO_2_, Partial pressure of oxygen/Fraction of inspired oxygen.

^a^ Cardiovascular disease is defined as a diagnosis of hypertension, ischemic heart disease, heart failure, atrial fibrillation, atrial flutter, peripheral vascular disease, and cerebrovascular disease.

^b^ Pulmonary disease is defined as asthma, chronic obstructive lung disease, and restrictive lung diseases.

^c^ Early time period as reference.

^d^ Highest level of respiratory support with the group supported by low flow oxygen or high flow nasal oxygen as reference.

When BMI was assessed for monotonicity and linearity using restricted cubic splines with four knots, there was an indication of non‐linearity (*p* = 0.08). Consequently, BMI was further explored through categorization with normal weight (20–25 kg/m^2^) as the reference. The odds ratios for BMI categories revealed a gradual decline in the risk of air‐leak syndrome with increasing BMI, with the highest risk observed in patients with a BMI below 20 kg/m^2^. However, when BMI was increased to 40 kg/m^2^, the risk was no longer seen to decline (Table 5). Also illustrated in Table 5, the mortality at day 90 by BMI category showed a nadir at BMI category 30–35 kg/m^2^ with an OR of 0.48 (95% CI 0.29–0.80) compared to normal weight.

**TABLE 5:** Results of univariable regression analyses of risk of air‐leak syndrome and mortality by body mass index category.

	No. of patients	Air‐leak syndrome		Mortality at day 90	
BMI category	661	OR	95% CI	OR	95% CI
<20 kg/m^2^	16	1.68	0.44–5.28	2.52	0.89–7.43
20 to <25 kg/m^2^	151	1.0 (ref)		1.0 (ref)	
25 to <30 kg/m^2^	235	0.71	0.40–1.27	0.78	0.50–1.22
30 to <35 kg/m^2^	172	0.51	0.26–1.00	0.48	0.29–0.80
35 to <40 kg/m^2^	61	0.35	0.10–0.96	0.82	0.42–1.55
≥40 kg/m^2^	26	0.66	0.15–2.08	1.03	0.42–2.45

*Note*: Results of the univariable analysis showing odds ratio between BMI category and the outcomes of air‐leak syndrome and mortality at day 90.

Abbreviations: BMI, body mass index; CI, confidence interval; OR, odds ratio.

## 4 DISCUSSION

In this study, we found that 12% of ICU patients with COVID‐19 were diagnosed with air‐leak syndrome, with the majority (72%) presenting with PMD. This aligns with prior COVID‐19 literature reporting air‐leak syndrome rates between 11 and 22%, with a high proportion manifesting as PMD.^8^, ^12^ Additionally, consistent with earlier studies, air‐leak syndrome was associated with a higher mortality compared to no air‐leak and data showed that 22% of air‐leak syndrome cases occurred without prior IV.^10^, ^11^, ^15^, ^16^, ^17^ When comparing the risk of air‐leak syndrome in ICU patients with respiratory failure due to COVID‐19 with literature reporting on air‐leak in ARDS patients without COVID‐19, the proportions of patients affected are described to be similar, between 6% and 11%.^3^, ^7^ However, if reported, the manifestation is predominantly PTX for non‐Covid‐19 patients. Finding stronger evidence of the true incidence of PMD in non‐COVID‐19 ARDS has proven challenging due to sparse literature. Nevertheless, this observation aligns with our results, suggesting that PMD was a relatively uncommon occurrence in ARDS patients before the COVID‐19 pandemic. This could imply that SARS‐CoV‐2 in itself causes another type of lung frailty which could cause air leakage outside the tracheobronchial tree, rather than a ventilator‐induced high transpulmonary pressure alone.^8^ In recent years, more attention has also been brought to the concept of patient self‐inflicted lung injury (P‐SILI). Patients believed to be at high risk of P‐SILI are those with already injured lungs and a high respiratory drive. This high respiratory drive can further harm the lungs by causing excessive transpulmonary pressure, resulting in over‐distension, inhomogeneous distribution of transpulmonary pressure variations across the lung, atelectotrauma, pendelluft phenomenon, and increased transvascular pressure resulting in pulmonary oedema.^16^ An earlier COVID‐19 study suggested that direct viral damage to the lungs, further exacerbated by P‐SILI, could increase the risk of air‐leak syndromes, both with and without the use of IV.^18^ To confirm these findings, conducting further research using comparable datasets on ARDS patients, both with and without COVID‐19, is essential. Recently, the definition of ARDS was updated to include patients using HNFO or even those without respiratory support in resource‐limited settings, reflecting the global realities observed during the pandemic.^19^ For COVID‐19 patients, the new definition has been shown to reduce controversy in diagnosing COVID‐19 ARDS. Furthermore, it underscores the significance of the new definition for COVID‐19 patients who met only the new criteria, as their ICU mortality rates were comparable to those meeting the old criteria, implying that they should receive equivalent supportive care.^20^, ^21^

In context of this discussion, it is, however, also important to note the strong association between IV and air‐leak syndrome in patients with COVID‐19. IV enables high pressure to be transmitted to the lungs and is also a signal of more severe disease requiring a higher level of respiratory support. Interestingly, although not statistically significant, patients treated with NIV prior to air‐leak syndrome, as compared to LFO/HFNO, had an OR of 0.23 (95% CI 0.05–1.05). This may indicate that too little respiratory support, resulting in P‐SILI, could also be a risk factor for air‐leak syndrome. The discussion about P‐SILI and its role in both non‐invasive and invasive respiratory support is ongoing, and much remains unknown.^22^

A number of other studies report that a concurrent burden of illness, especially underlying pulmonary disease and cardiovascular conditions are clearly associated with air leaks.^11^, ^23^ In agreement with these previous studies, we found pulmonary disease at baseline to be associated with an increased risk of air‐leak syndrome, however, no association was found between cardiovascular disease at baseline and air‐leak.

We observed a consistent, gradual decrease in the risk of air‐leak syndrome with increasing BMI, up to 40 kg/m^2^, confirming the findings of a prior COVID‐19 study.^11^ This is not the first time a high BMI has been associated with a protective effect in ICU patients. When BMI has been investigated, a high BMI has even shown a decreased mortality, referred to as the “ICU obesity paradox.”^25^, ^26^, ^27^ The complexity behind the obesity paradox is very difficult to untangle and is beyond the scope of this study, even if we saw this trend also for 90‐day mortality, Table 5. Nonetheless, an association between BMI and air‐leak may be understandable physiologically, with weight from the thorax and abdomen increasing the transthoracic pressure and thereby protecting the lung from overdistension and high transpulmonary pressure.^18^, ^24^ Whether a decreased risk of air‐leak syndrome is one of the protective reasons for a high BMI being associated with lower mortality among ICU patients is speculative. That said, the data forming the obesity paradox is mainly from patients cared for prior to the pandemic. For COVID‐19 patients, obesity has been established as a risk factor for ICU admission, but once admitted to the ICU, obesity may no longer be a risk factor. A large Swedish study from the beginning of the pandemic could not show any association between BMI and mortality in the ICU, and neither could a meta‐analysis of more than 6000 patients.^28^, ^29^ Increased mortality with higher BMI was indeed shown in one registry‐based study, but when the results were broken down into BMI categories, a significant increase in the composite outcome of length of stay and mortality was only seen in patients with a BMI of more than 30 kg/m^2^ and even 35 kg/m^2^ for some of the models.^30^ To confirm whether there is an obesity paradox also for ICU patients with COVID‐19, further research is needed, and BMI should probably not be handled as a linear variable in such studies.

A higher proportion of patients diagnosed with air‐leak syndrome were proned, and we speculate that this reflects a higher severity of illness in these patients rather than a direct relationship between air‐leak syndrome and treatment with proning.

A prominent result in our study was the higher risk of air‐leak syndrome in the later compared to the early time period. Early in the pandemic, air‐leak syndrome might have been underdiagnosed due to a heavy workload and fear of viral contamination during transport to radiology departments before we had developed safe routines for preforming diagnostics.

Another difference between the two time periods was the introduction of glucocorticoids. At Södersjukhuset, glucocorticoids became the standard of care for all COVID‐19 patients admitted to the ICU shortly after the RECOVERY trial presented preliminary results in a press release on June 16, 2020, demonstrating reduced mortality in invasively ventilated COVID‐19 patients.^14^, ^31^ The impact of these results is clearly visible in Table 3, illustrated by the higher proportion of patients with glucocorticoid therapy in the later, compared with the early time period. Due to this collinearity, we did not include glucocorticoids in our model. We chose to focus on time periods rather than glucocorticoids to reflect the evolving understanding of the disease, which led to changes in care, diagnostics and treatment, as glucocorticoids was just one aspect of these changes. But it is worth mentioning that other observational studies have found an association between glucocorticoid therapy and the likelihood of air‐leak syndrome manifestations in both non‐ventilated and ventilated COVID‐19 patients, prompting speculation that glucocorticoids may contribute to the weakening of the alveolar wall and thereby increasing the risk of air‐leak syndrome.^32^, ^33^ If this finding truly has a causal relationship, it is still difficult to see how it would impact care, as an increased chance of survival outweighs a higher risk of air‐leak syndrome.

## 5 LIMITATIONS

Our study had several limitations.

First, the retrospective nature carries a risk for confounding and, therefore, only hypothesis‐ generating associations can be established. One important confounder is the temporal change in care of ICU patients with COVID‐19, both with regards to treatment given and investigations made. We have tried to adjust results by including time of admission (time period) when analysing potential associated variables.

Second, we have not analysed data regarding ventilation mode, airway pressure, or length of ventilator support. Hospital guidelines strongly recommend lung‐protective ventilation according to ARDS guidelines, using tidal volumes of 6–8 mL/kg ideal body weight, maintaining plateau pressures below 30 cm H₂O, using prone positioning for patients requiring 60% or more oxygen, and titrating positive end‐expiratory pressure (PEEP) to achieve the lowest possible driving pressure.^3^, ^5^, ^34^, ^35^ However, as practising clinicians, we know that patients sometimes experience periods where it is difficult to adhere to lung‐protective ventilation due to severe hypoxia and/or hypercarbia. To manage this, patients were often exposed to multiple ventilatory modes while searching for the most lung‐protective option, making it very challenging to retrospectively categorize patients based on exposure to specific modes. We do acknowledge that ventilatory support strategies probably differed between time periods. For example, in the early phase, there was a higher intubation rate, greater utilization of anaesthesia machines instead of intensive care ventilators, and a reliance on more basic ventilatory modes (volume‐ and pressure‐controlled ventilation). In contrast, the later period saw an increased use of advanced modes such as airway pressure release ventilation. These differences in ventilatory management may partially explain the variation in air‐leak syndrome risk between the early and later time period, as one of many temporal changes in treatment practices. Furthermore, we could not establish specific thresholds for airway pressures or the duration of ventilatory support to categorize ventilator treatment exposure accurately. These variables are likely critical for understanding the risk of developing air‐leak syndrome. However, investigating the outcomes of different ventilatory strategies would likely require a prospective interventional study to separate group exposures effectively.

Third, we do not have the exact time point for when the air‐leak developed as CXR and CT were only preformed at physicians' discretion. We have chosen to use the time point of the confirming CXR/CT no matter if it was a clinical suspicion of air‐leak or if it was an incidental finding. Hence, the temporal uncertainty is important to consider when making associations with other variables changing over time, as for us, when analysing the level of respiratory support. For nine of the patients diagnosed with air‐leak syndrome with IV as their highest level of respiratory support, we cannot confirm whether the syndrome had already developed prior to intubation. This uncertainty arises because no chest CXR or CT without signs of air‐leak was available after the placement of the endotracheal tube but prior to the air‐leak diagnosis. For the remaining patients, this information could be confirmed. We therefore conducted a sensitivity analysis excluding these nine patients to avoid overestimating the association between air‐leak syndrome and invasive ventilation. This exclusion did not change the results for IV or any other variables in the multivariable regression (Table 4).

Fourth, due to the retrospective design, we have missing data as the heavy workload during the pandemic made data registration difficult. However, only a very small proportion of the variables of BMI (1%, 8/669) and PaO_2_/FiO_2_ (0.5%, 3/669) were missing and these patients were excluded from the analysis of variables associated to air‐leak syndrome.

## 6 CONCLUSION

In 669 ICU patients with COVID‐19, 12% had one or more manifestations of air‐leak syndrome. Notably, PMD, rather than PTX, was the most common manifestation, suggesting a potentially distinctive feature of COVID‐19‐related air‐leak syndrome. Further research is needed to determine whether COVID‐19 involves different pathophysiological or iatrogenic mechanisms compared with other critical respiratory conditions.

## AUTHOR CONTRIBUTIONS

A. Löwing Jensen, J. Litorell, M. Dahlberg, E. Joelsson‐Alm, and S. Jonmarker conceptualized the study and the methodology. A. Löwing Jensen, J. Litorell, M. Dahlberg, E. Joelsson‐Alm, and S. Jonmarker manually extracted data from electronic health records. A. Löwing Jensen, J. Litorell, E. Joelsson‐Alm, and S. Jonmarker were responsible for choosing definitions. S. Jonmarker performed the statistical analyses and the visualizations of the results. A. Löwing Jensen and S. Jonmarker wrote the manuscript, which all other authors revised critically for intellectual content and then approved for submission.

## Data Availability

The data that support the findings of this study are available on request from the corresponding author. The data are not publicly available due to privacy or ethical restrictions.
